# Identification of repurposed drugs targeting significant long non-coding RNAs in the cross-talk between diabetes mellitus and Alzheimer’s disease

**DOI:** 10.1038/s41598-022-22822-9

**Published:** 2022-10-31

**Authors:** Shokoofeh Ghiam, Changiz Eslahchi, Koorosh Shahpasand, Mehran Habibi-Rezaei, Sajjad Gharaghani

**Affiliations:** 1 Laboratory of Bioinformatics and Drug Design, Institute of Biochemistry and Biophysics, University of Tehran, Tehran, Iran; 2 Department of Computer and Data Sciences, Faculty of Mathematical Sciences, Shahid Beheshti University, Tehran, Iran; 3 School of Biological Sciences, Institute for Research in Fundamental Sciences (IPM), Tehran, Iran; 4 Department of Brain and Cognitive Sciences, Cell Science Research Center, Royan Institute for Stem Cell Biology and Technology, ACECR, Tehran, Iran; 5 School of Biology, College of Science, University of Tehran, Tehran, Iran

**Keywords:** Computational biology and bioinformatics, Systems biology, Biomarkers, Diseases

## Abstract

The relationship between diabetes mellitus (DM) and Alzheimer’s disease (AD) is so strong that scientists called it “brain diabetes”. According to several studies, the critical factor in this relationship is brain insulin resistance. Due to the rapid global spread of both diseases, overcoming this cross-talk has a significant impact on societies. Long non-coding RNAs (lncRNAs), on the other hand, have a substantial impact on complex diseases due to their ability to influence gene expression via a variety of mechanisms. Consequently, the regulation of lncRNA expression in chronic diseases permits the development of innovative therapeutic techniques. However, developing a new drug requires considerable time and money. Recently repurposing existing drugs has gained popularity due to the use of low-risk compounds, which may result in cost and time savings. in this study, we identified drug repurposing candidates capable of controlling the expression of common lncRNAs in the cross-talk between DM and AD. We also utilized drugs that interfered with this cross-talk. To do this, high degree common lncRNAs were extracted from microRNA-lncRNA bipartite network. The drugs that interact with the specified lncRNAs were then collected from multiple data sources. These drugs, referred to as set D, were classified in to positive (D^+^) and negative (D^−^) groups based on their effects on the expression of the interacting lncRNAs. A feature selection algorithm was used to select six important features for D. Using a random forest classifier, these features were capable of classifying D^+^ and D^−^ with an accuracy of 82.5%. Finally, the same six features were extracted for the most recently Food and Drug Administration (FDA) approved drugs in order to identify those with the highest likelihood of belonging to D^+^ or D^−^. The most significant FDA-approved positive drugs, chromium nicotinate and tapentadol, were presented as repurposing candidates, while cefepime and dihydro-alpha-ergocryptine were recommended as significant adverse drugs. Moreover, two natural compounds, curcumin and quercetin, were recommended to prevent this cross-talk. According to the previous studies, less attention has been paid to the role of lncRNAs in this cross-talk. Our research not only did identify important lncRNAs, but it also suggested potential repurposed drugs to control them.

## Introduction

Recent studies^1,2^ have linked insulin resistance (IR) and impaired insulin signaling to the cross-talk between diabetes mellitus (DM) and Alzheimer’s disease (AD). Many scientists believe that Alzheimer's disease is both a neurological and neuroendocrine disorder. Therefore, Type 3 Diabetes (T3D) is the most appropriate term to describe this complicated disease^3^. Other cellular and molecular factors involved in the cross-talk between DM and AD are oxidative stress, obesity, inflammation, dysregulation of Apolipoprotein E (APOE), Insulin Degradation Enzyme (IDE), Glucose Transporter type 4 (GLUT4), and Acetylcholinesterase (AChE)^4^. Nowadays, computational-based methods are very popular because they save time and money, so experts are using computational methods to introduce important genes, proteins, drugs etc.

To overcome this cross-talk, various computational-based studies have been performed so far. Mittal et al. extracted differentially expressed genes from Type 2 diabetes mellitus (T2DM) and Alzheimer’s patients. Then, they constructed a protein–protein interaction network using the proteins' first neighbors. Pathway enrichment analysis was also performed, and notable proteins as well as significant pathways were introduced^5^.

Using Biological Expression Language, Karki et al.^6^ proposed six shared pathways between T2DM and AD. They also discussed the dangers of certain anti-diabetic drugs in the progression of AD.

Hu et al.^7^ employed data from the ROSMAP Project to introduce common pathways between T2DM and AD. They analyzed multi-omic data using an inference method.

In addition to the importance of proteins, recent research suggests that non-coding RNAs (ncRNAs) play an important role in the progression of complex diseases^8–11^. The reason is that they are encoded by the majority of the human genome. Furthermore, approximately two-thirds of the experimentally detected microRNAs (miRNAs) are expressed in the brain and are active participants in its functions^12^. Long non-coding RNAs (lncRNAs) have also been shown to play a biomarker role in the detection of IR, inflammation, and DM, as well as the progression of AD^13,14^.

Ghiam et al.^15^ investigated the role of the ncRNAs as potential biomarkers in the cross-talk between DM and AD.

In addition to the biomarker role of ncRNAs in early disease detection, they may also be used as pharmacological targets in the treatment of a variety of disorders^16^. On the other hand, a new drug takes a long time to complete all phases of drug design (at least 10 years). This procedure is both time-consuming and expensive. To address the aforementioned problems, computer-aided drug design (CADD) has been proposed as a method for predicting new drugs for further study. Methods of drug repositioning (also known as drug repurposing) have been utilized to identify new therapeutic roles for existing drugs. For this purpose, Shakil used molecular docking to investigate the effects of two antidiabetic drugs, Ertugliflozin and Sotagliflozin, and their targets, Sodium glucose cotransporter 2 and AChE, on AD. Finally, he emphasized the sotagliflozin structure for future Type 3 diabetes treatment^17^.

This study aims to introduce (reuse) Food and Drug Administration (FDA) approved drugs for regulating the expression of common lncRNAs in the cross-talk between DM and AD. Additionally, drugs with side effects were reported.

The following section discusses data collection, machine learning, and network-based methods. Positive drugs (candidates for repurposing) and negative drugs (those with adverse effects) were proposed in the results section, and we will elaborate on them in the discussion section.

## Materials and methods

### Materials

#### Collection of data

Insulin is required for normal cognitive functions of the hippocampus. According to scientists, the primary factor in the cross-talk between DM and AD is insulin resistance in the brain^18^. Consequently, significant mRNAs were extracted from DM, AD, and IR. The set of common mRNAs was selected as follows:

$${\text{P}} = {\text{DM}}_{{\text{p}}} \cap {\text{AD}}_{{\text{p}}} \cap {\text{IR}}_{{\text{p}}}$$

where DM_p_, AD_p_ and IR_p_ denote the sets of curated proteins associated with DM, AD and IR in the Disgenet database respectively.

The information of miRNAs and lncRNAs were collected from miRTarBase^19^, LncBase^20^, and Starbase^21^ databases. We attempted to select the most reliable mRNA–miRNA, miRNA–lncRNA, and lncRNA–drug interactions with experimental evidence. To do so, we looked for mRNA–miRNA and miRNA–lncRNA interactions in the Starbase database with CLIP-Data ≥ 3 (strict selection), mRNA–miRNA interactions in the miRTarBase database with strong evidence, and miRNA–lncRNA interactions in the LncBase database with high confidence. These interactions are chosen to be functional with the best binding capabilities.

The lncRNA-drug information was gathered from the D-lnc^22^ and NancoRNA^23^ databases. Finally, the information of the latest FDA approved drugs with the structural files were downloaded from DrugBank^24^.

### Method

**Figure 1:**
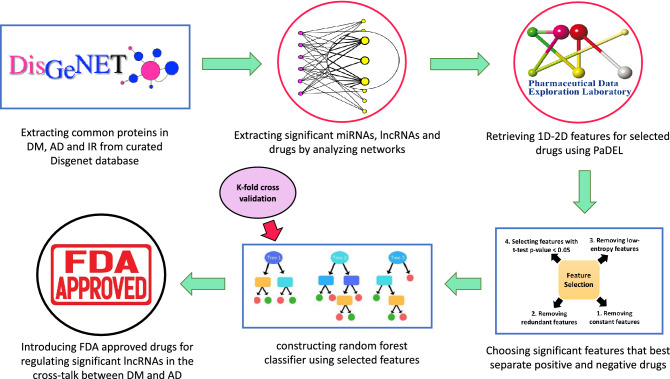
The workflow of the study.

We extracted common mRNAs in DM, AD, and IR from the curated Disgenet database in the first step. After retrieving miRNAs and lncRNAs from experimentally detected databases for the selected mRNAs, high degree miRNAs and lncRNAs were extracted from mRNA–miRNA and miRNA–lncRNA networks, respectively. Following that, for the significant lncRNAs, drug-lncRNA interactions were extracted from various datasets, and drugs were classified into positive and negative groups based on their effects on the regulation of the selected lncRNAs. To build a model for selecting FDA-approved drugs for repurposing, one-dimensional and two-dimensional (1D-2D) features of the intended drugs were extracted, and the feature selection algorithm was used to select features that could best separate positive and negative sets. On the basis of the selected features, the random forest classifier was then constructed, and performance metrics were reported. Finally, using a constructed random forest model, the probability of each FDA-approved drug belonging to positive or negative sets was predicted, and the most relevant drugs to each set were introduced by defining a threshold. Those in the positive group were proposed as potential candidates for repurposing to regulate important lncRNAs, whereas those in the negative group were proposed as adverse drugs in the cross-talk between DM and AD. Figure 1 shows the workflow of the study.

In the following, each step has been described in detail.

#### Identification of significant miRNAs based on the mRNA-miRNA network

A mRNA–miRNA bipartite network was built in the first state. The nodes in one part of this network represent a set of selected mRNAs, while the nodes in the other part represent miRNAs which have interacted with at least one of the selected mRNAs retrieved from databases. Assume that P = {p_1_, p_2_, …, p_x_} and M = {m_1_, m_2_, …, m_l_} represent the set of mRNAs and miRNAs, respectively. If miRNA m_j_ interacts with mRNA p_i_, there is an edge between p_i_ and m_j_.

High degree miRNAs were chosen as significant in the constructed network. These miRNAs are denoted by M′ = {m′_1_, m′_2_, m′_3_, …, m′_s_}.

#### Identification of significant lncRNAs based on the miRNA-lncRNA network

In this state, the miRNA-lncRNA bipartite network was constructed. Nodes in one part are the set of significant miRNAs (M′) and nodes in the other part are the set of lncRNAs received from databases which have interacted with at least one of the selected miRNAs. Assume that L = {l_1_, l_2_, …, l_r_} is the set of mentioned lncRNAs. If lncRNA l_j_ interacts with miRNA m'_i_, there is an edge between m'_i_ and l_j_. High degree lncRNAs were chosen as significant in this network. These lncRNAs are denoted by L′ = {l′_1_, l′_2_, l′_3_, …, l′_t_}.

#### Drug collection based on the lncRNA-drug network

At first, the regulation (upregulation or downregulation) effect of members of L′ in both DM and AD were diagnosed. Let D = {d_1_, d_2_, d_3_, …, d_z_} denoted the set of drugs which interacted with at least one of the elements in L′. Suppose that d_i_ interacts with both l′_i_ and l′_j_ which are upregulated and downregulated respectively in both DM and AD. It means that d_i_ inhibits both of them therefore, the effect of d_i_ on l′_i_ is positive (therapeutic effect) but on l′_j_ is negative (exacerbation effect). In this case we remove d_i_ from D. Accordingly, drugs with contradictory effect were omitted. Let D′ = {d′_1_, d′_2_, d′_3_, …, d′_n_} are denoted the set of remaining drugs. Now we constructed the lncRNA-drug bipartite network which nodes in one part are L′ and the other part are D′. There is an edge between l′_i_ and d′_j_ if drug d′_j_ interacts with lncRNA l′_i_. Next, based on the therapeutic effect or side effect of D′ on the regulation of members in L′, D′ was divided in to D^+^ = {d′_1_, …, d′_j_} which cure the intended diseases and D^−^  = {d′_j+1_, …, d′_n_} which severely increase the negative effects of the intended diseases. For instance, suppose that l′_i_ is upregulated in both DM and AD and d′_i_ inhibits it therefore, d′_i_ will be categorized in D^+^. On the other hand, suppose that l′_j_ is upregulated too but d′_k_ causes overexpression of this lncRNA (inverse effect) so, by using this drug, the negative effects of both DM and AD will increase. As a result, d′_k_ will be categorized in D^−^.

The regulation (upregulation or downregulation) effect of L′ members in both DM and AD was initially identified. D = {d_1_, d_2_, d_3_, …, d_z_} represents the set of drugs that interacted with at least one element in L′.

Suppose that d_i_ interacts with l′_i_ and l′_j_, which are respectively upregulated and downregulated in both DM and AD. It indicates that d_i_ inhibits both l′_i_ and l′_j_ therefore, the effect of d_i_ on l′_i_ is beneficial (therapeutic effect), while its effect on l′_j_ is detrimental (exacerbation effect). In this instance, we eliminate d_i_ from D. Thus, drugs with contradictory effects were excluded. Let D′ = {d′_1_, d′_2_, d′_3_, …, d′_n_} represents the remaining drugs. Now we have constructed the lncRNA-drug bipartite network, with L′ nodes in one part and D′ nodes in the other. If d′_j_ interacts with lncRNA l′_i_, there is an edge between l′_i_ and d′_j_. Next, based on the therapeutic effect or side effect of D′ on the regulation of members in L′, D′ was divided into D^+^  = {d′_1_, …, d′_j_} which cure the intended diseases and D^−^  = {d′_j+1_, …, d′_n_} which significantly intensify the negative effects of the intended diseases. For example, if l′_i_ is upregulated in both DM and AD and d′_i_ inhibits it, then d′_i_ will be classified as D^+^. Alternatively, if l′_j_ is also upregulated but d′_k_ causes overexpression of this lncRNA, then the use of this drug will exacerbate the negative effects of both DM and AD. Consequently, d′_k_ will be classified as D^−^.

#### Features of drugs

For drugs in D′, we converted sdf files to mol files for using Open Babel^25^, a tool for converting chemical file formats. PaDEL^26^, a software for calculating molecular descriptors and fingerprints, was also employed to extract 1D–2D features based on the structural properties of D′ members. Only those features that best classified D′ into D^+^ and D^-^ were selected. The algorithm for feature selection is as follows:

1. Eliminating constant features: Features that have the same value for all drugs in D' were omitted because they had no impact on the classification process.
2. Removing redundant features: To improve performance, highly correlated features should be removed. To achieve this, we computed the Pearson correlation coefficient using the "findcorrelation" function from the "caret" package in R, with a cutoff of 0.9. This function takes the absolute values of pair-wise correlations into account. The function eliminates the variable with the highest mean absolute correlation when two variables have a high correlation.
3. Discarding features with low entropy: To find efficient variables for the model, entropy was calculated for each feature using the entropy function in R, and features with entropy less than θ were removed.
4. Removing features with a p-value greater than 0.05: For the remaining attributes, the t-student test was used to select features with significant differences (*p* value < 0.05) between D^+^ and D^−^.

Finally, significant attributes F = {f_1_, f_2_, f_3_, …, f_w_} were chosen

#### Model designing

A random forest classifier was constructed using features extracted from the previous state.

Out of bagging error (OOB) was calculated using Python's random forest library to determine the optimal number of trees. OOB is a type of random forest mean prediction error in which trees without the intended sample in their bootstrap are utilized. The model was then evaluated using five-fold cross validation, and performance metrics were reported.

#### Choosing FDA-approved drugs that are relevant to members of D′

In the same manner as in previous states, PaDEL was used to extract 1D-2D features for FDA-approved drugs, and the same set of extracted features, F, was selected. Let D_FDA_ = {df_1_, df_2_, df_3_, …, df_v_} denote drugs with selected features. Then, for DFDA, outlier detection was utilized to select the most pertinent FDA-approved drugs for D*′*.

The procedure for detecting outliers is as follows:

1. Number of atoms: very small drugs with nATOM ≤ µ was removed from the study since they have no impact on the classification process.
2. Applicability of domain: the warning leverage for each compound was calculated using the formula: h_i_ = x_i_^T^ (X^T^ X)^−1^ x_i_
  where:
3. x_i_ is the query molecule's description-row vector
4. X is the k*n matrix (k descriptor values for each one of the n training molecules)
5. h* (warning leverage) is usually set to 3p/n, where p is the number of model variables (|F|) plus one and n is the number of training compounds
  Outliers were identified as drugs with a h_i_ value greater than the warning leverage and were removed from the dataset.
6. One class classification: although relevant drugs were selected from previous steps by removing outliers, to further optimize the running time of the classification algorithm, it is necessary to select highly relevant drugs to D′. To achieve this, for the remaining drugs in the D_FDA_, a single class classification method employing Support Vector Machine (SVM) classifier with radial basis^27^ was utilized, and other outliers were excluded.

Finally, the most relevant compounds to D' were chosen from all FDA-approved drugs. These drugs are denoted by D′_FDA_ = {df′_1_, df′_2_, df′_3_, …, df′_q_}. Now, this set was fed into the newly constructed random forest model, and for each drug the probability of belonging to D^+^ or D^−^ was calculated. The drugs with probabilities of 0.95 or higher were selected. Those with a positive effect (belongs to D^+^) were suggested as repurposing candidates and those with a negative effect (belongs to D^−^) were introduced as warning drugs that could simultaneously increase the risk of DM and AD.

## Results

### Network analysis

**Figure 2:**
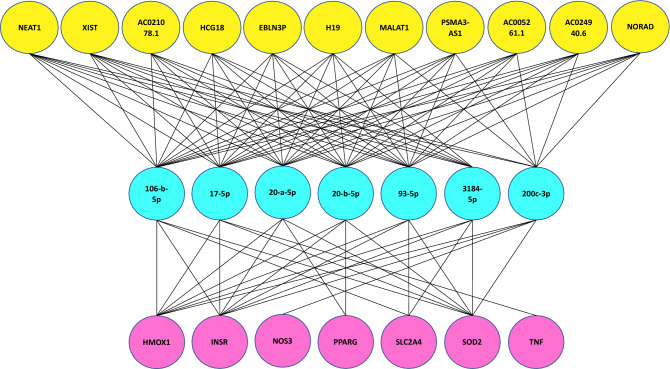
Final mRNA-miRNA-lncRNA network. The first layer (purple nodes) consists of 7 shared mRNAs between DM and AD that were extracted from curated Disgenet database. The second layer (blue nodes) consists of 7 high degree miRNAs obtained from mRNA-miRNA bipartite network and the last layer (yellow nodes) consists of 11 high degree lncRNAs achieved from miRNA-lncRNA bipartite network.

Based on the data collection section above, 9 mRNAs shared by DM, AD, and IR were extracted from the curated Disgenet database (Table 1, first column). There are 412 miRNAs which have interacted with at least one of these mRNAs. Then we constructed the mRNA-miRNA bipartite network and significant miRNAs were extracted. In this network, no miRNA interacts with the INS and LEP proteins, so these two proteins were omitted from our investigation. Then, miRNAs with the highest degree (degree 4) were chosen as significant. By this strategy, 7 miRNAs were identified (Table 1, second column). Similarly, we constructed the miRNA-lncRNA bipartite network. This network contains 1700 lncRNAs, only three of them have the highest degree of 7, and eight of them have degree 6. These 11 lncRNAs were deemed significant (Table 1, forth column). These degrees are chosen so that approximately 10 miRNAs and 10 lncRNAs are considered as significant. For instance, if we considered degree 3 for the first network, we would select more than 40 miRNAs, and if we considered degree 5 for lncRNAs, we would select more than 30 lncRNAs. Supplementary Tables S1 and S2 list all mRNA-miRNA and miRNA-lncRNA interactions in these two networks. However, the final tripartite network was built using 7 selected mRNAs, 7 selected miRNAs, and 11 selected lncRNAs. Results have been shown in Fig. 2 and Table 1.”

**Table 1:** Selected mRNAs, miRNAs and lncRNAs.

MRNAs (P)	MiRNAs (M′)	Degree	LncRNAs (L′)	Degree
INSR	hsa-miR-3184-5p	4	NEAT1	7
NOS3	hsa-miR-17-5p	4	XIST	7
PPARG	hsa-miR-20a-5p	4	EBLN3P	7
SLCA4	hsa-miR-93-5p	4	H19	6
SOD2	hsa-miR-106b-5p	4	MALAT1	6
HMOX1	hsa-miR-20b-5p	4	HCG18	6
TNF	hsa-miR-200c-3p	4	PSMA3-AS1	6
INS	–		AC021078.1	6
LEP	–		AC005261.1	6
			AC024940.6	6
			NORAD	6

Significant mRNAs, miRNAs, and lncRNAs are summarized in Table 1. No interacting miRNAs have been reported in the existing databases for INS and LEP among the 9 mRNAs. The hub miRNAs and lncRNAs are shown in columns 2 and 4, and the associated degrees of the intended miRNAs and lncRNAs are shown in columns 3 and 5.

Significant mRNAs, miRNAs, and lncRNAs are summarized in Table 1. No interacting miRNAs have been reported in the existing databases for INS and LEP among the 9 mRNAs. The hub miRNAs and lncRNAs are shown in columns 2 and 4, and the associated degrees of the intended miRNAs and lncRNAs are shown in columns 3 and 5.

To validate the role of obtained mRNAs in the cross-talk between DM and AD, their dysregulation’s type was investigated. The literature review indicates that HMOX1, NOS3, PPARG, and TNF are upregulated in both diseases, while INSR, SLC2A4, and SOD2 are downregulated. The formation of neurofibrillary tangles is caused by the upregulation of HMOX1, which increases tau hyperphosphorylation and results in neurofibrillary tangle formation^28^. Overexpression of PPARG causes amyloid beta (Aβ) neurotoxicity by targeting glycogen synthase kinase-3beta (GSK3β) in the Wnt/Beta-Catenin pathway. The upregulation of NOS3 and TNF may increase apoptosis^29^ and inflammation^30^ in the memory region of the brains of patients with DM and AD, respectively. Conversely, downregulation of INSR reduces insulin's sensitivity to its receptors, resulting in insulin resistance in the brain^31^. SLC2A4 (GLUT4) is the brain's glucose transporter. Any dysregulation of the expression of this important protein may impair neuronal cells' ability to absorb glucose^32^. SOD2 downregulation also affects mitochondrial activity in the neuronal cells^33^. Summary of the results are presented in Table 2.

**Table 2:** mRNAs interpretation.

mRNA	Expression	Mechanism
HMOX1	Upregulation	Formation of neurofibrillary tangles
INSR	Downregulation	Insulin resistance
NOS3	Upregulation	Apoptosis in cortical neurons
PPARG	Upregulated	Targeting Wnt/Beta-Catenin Pathway
SLC2A4	Downregulation	Disrupting glucose transportation in the brain
SOD2	Downregulation	Mitochondrial dysfunction
TNF	Upregulation	Increasing inflammation

In this table, 7 mRNAs in the cross-talk between DM and AD (achieved from curated Disgenet database) and their mechanisms in the relationship between the two intended diseases are introduced using literature review

In this table, 7 mRNAs in the cross-talk between DM and AD (achieved from curated Disgenet database) and their mechanisms in the relationship between the two intended diseases are introduced using literature review.

For the 7 significant miRNAs identified by the miRNA-lncRNA bipartite network, we also conduct a literature review to confirm their role in the cross-talk between DM and AD.

In both diseases, hsa-miR-106b-5p, hsa-miR-17-5p, hsa-miR-20a-5p, hsa-miR-93-5p, and hsa-miR-200c-3p have been downregulated, whereas hsa-miR-20b-5p has been upregulated. Downregulation of hsa-miR-106b-5p, hsa-miR-17-5p, has-miR-20a-5p and hsa-miR-93-5p as well as overexpression of hsa-miR-20a-5p may alter the expression of amyloid precursor protein (APP) and subsequently influence Aβ, which is responsible for hyperphosphorylation of tau protein. In addition, altering the expression of hsa-miR-200c-3p may increase the expression of GSK3β, which results in hyperphosphorylation of tau^34^. Moreover, hsa-miR-3184-5p is a novel miRNA in the cross-talk between DM and AD, which is proposed in this study for future experimental investigation. Table 3 shows the summary of these results.

**Table 3:** miRNAs interpretation.

miRNA	Expression	Mechanism
hsa-miR-106b-5p	Downregulation	Regulating Aβ induced tau phosphorylation via regulating the expression of Fyn
hsa-miR-17-5p has-miR-20a-5p	Downregulation	Regulating APP expression
hsa-miR-20b-5p	Upregulation	Attenuating apoptosis induced by Aβ_25–35_
hsa-miR-93-5p	Downregulation	Targeting Aβ plaques
hsa-miR-200c-3p	Downregulation	Increasing tau phosphorylation (p-tau) by increasing p-GSK-3β
hsa-miR-3184-5p	Novel miRNA	–

This table describes the mechanisms of 7 significant miRNAs in the cross-talk between DM and AD. Only miR-3184-5p introduced in this study as a novel miRNA in this cross-talk which needs additional experimental research.

This table describes the mechanisms of 7 significant miRNAs in the cross-talk between DM and AD. Only miR-3184-5p introduced in this study as a novel miRNA in this cross-talk which needs additional experimental research.

Similarly, we investigated the role of obtained lncRNAs in DM and AD cross-talk through literature review. Recent studies suggest that NEAT1 and XIST, two important biomarkers in DM and AD, are upregulated in both diseases, which not only activate the Akt/mTOR signaling pathway but also dysregulate the expression of miR-124, resulting in an increase in BACE1 concentration and Aβ plaque deposition in the brain^35–37^. H19, another significant lncRNA, is upregulated in DM and AD, resulting in hippocampal neuron apoptosis^38^. Meanwhile, low levels of MALAT1 (downregulation) have been found in Alzheimer’s patients compared to controls, reducing the lncRNA’s anti-apoptotic effect^39^. Abdulle et al. also demonstrated the significance of MALAT1 as a target in diabetic-related diseases^40^. Additionally, HCG18 and BPLN3P are upregulated, which increases the risk of insulin resistance and type 1 diabetes, respectively^41,42^. The remaining obtained lncRNAs are novel in this cross-talk which are proposed in this study for future experimental analysis. Table 4 displays the summary of these results.

**Table 4:** LncRNAs interpretation.

lncRNA	Expression	Mechanism
NEAT1	Upregulation	Biomarkers in DM and AD
XIST		
HCG18	Upregulation	Insulin resistance
EBLN3P	Upregulation	Increasing the risk of type 1 diabetes mellitus
H19	Upregulation	Apoptosis of hippocampal neurons
MALAT1	Downregulation	
PSMA3-AS1	Novel	–
AC021078.1	Novel	–
AC005261.1	Novel	–
AC024940.6	Novel	–
NORAD	Novel	–

This table explains the role of the obtained lncRNAs in the cross-talk between DM and AD. Among 11 significant lncRNAs, 5 of them are novel, proposed in this study for future experimental analysis

This table explains the role of the obtained lncRNAs in the cross-talk between DM and AD.

Among 11 significant lncRNAs, 5 of them are novel, proposed in this study for future experimental analysis.

Additionally, 40 drugs were extracted from lncRNA-drug bipartite network where 24 drugs classified as D^−^ and 16 drugs as D^+^. These drugs interact with NEAT1, XIST, H19 and MALAT1. For example, Panobinostat increases NEAT1 and H19 expression in SK-NEP-1 and G401 cells^43^ According to Table 4, the upregulation of the targeted lncRNAs may cause cross-talk between DM and AD. Consequently, the use of this drug may increase the risk of both DM and AD. As a result, it is classified as D^−^. In contrast, Adriamycin inhibits NEAT1 expression in stomach cancer. In addition, it can increase MALAT1 expression in non-small cell lung cancer. It may therefore have a therapeutic effect on the cross-talk between DM and AD and is classified as D^+^.

The remaining 7 lncRNAs did not interact with any drugs in the existing lncRNA-drug databases. Table 5 displays the results of the negative or positive effect of all the obtained drugs on the expression of the 4 selected lncRNAs.

**Table 5:** Drugs and the corresponding lncRNAs.

Drugs (D^−^)	lncRNAs	Drugs (D^+^)	lncRNAs
Panobinostat	NEAT1, H19	Adriamycin	NEAT1, MALAT1
Diamorphine	NEAT1	Dexamethasone	NEAT1
Deferasirox	NEAT1	Arachidonic acid	NEAT1
Ubiquinol	NEAT1	Sorafenib	NEAT1
Nilotinib	NEAT1, XIST	Estradiol	NEAT1
Rosiglitazone	NEAT1	Clinafloxacin	NEAT1
Trovafloxacin	NEAT1	Fluorouracil	NEAT1
Sangivamycin	NEAT1, XIST	Oxymatrine	MALAT1
Bisphenol A	NEAT1	Quercetin	XIST, MALAT1
Apratoxin A	NEAT1, XIST	pma	MALAT1
Bexarotene	NEAT1	Curcumin	H19
beta-Asarone	MALAT1	Plx4720	H19
Bleomycin	MALAT1	Mk-886	H19
3–3′–4 4′ tetrachlorobiphenyl	MALAT1	Tamoxifen	H19
Oxaliplatin	H19, MALAT1	Fulvestrant	H19
Vincristine	H19, MALAT1	Diclofenac	XIST
Etanercept	H19		
Doxycycline	H19		
Azathioprine	H19		
Decitabine	XIST, H19		
Etoposide	H19		
Verapamil	H19		
Epirubicin	H19		
Pirarubicin	H19		
Bortezomib	H19		
Azacitidine	XIST		

The drugs in the first column (D-) and the third column (D+) have a negative and positive effect on the common significant lncRNAs in DM and AD respectively. Furthermore, the interaction of lncRNAs with drugs is reported in columns 2 and 4, respectively.

The drugs in the first column (D^−^) and the third column (D^+^) have a negative and positive effect on the common significant lncRNAs in DM and AD respectively. Furthermore, the interaction of lncRNAs with drugs is reported in columns 2 and 4, respectively.

#### Feature description

**Figure 3:**
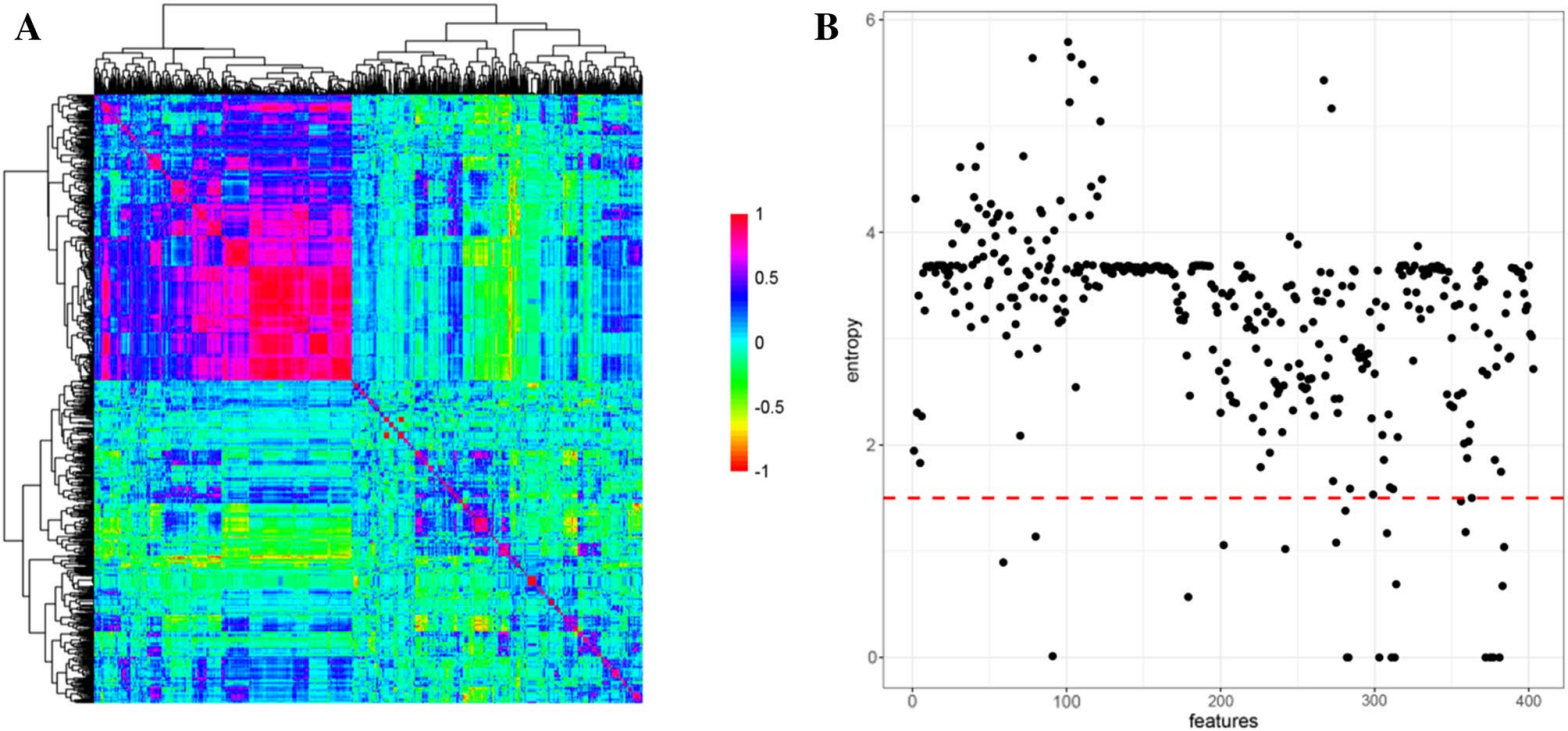
The feature selection process. (**A**) The correlation matrix of 1444 features achieved from PaDEL has been displayed. The values range from − 1 to 1, with − 1 denoting a negative correlation and 1 denoting a positive correlation. Features that were highly correlated (cutoff = 0.9) were removed. (**B**) Features with low entropy (below the red dashed line) were omitted.

PaDEL was utilized to extract 1444 features from the 40 drugs that were chosen from the lncRNA-drug bipartite network. There were 293 omitted constant features. Then, 745 highly correlated features with cutoff = 0.9 were also removed (Fig. 3A). Low entropy features (θ < 1.5) that had no effect on classifying D^+^ and D^−^ were removed as well (Fig. 3B).

Consequently, 293 features remained. The *t*-student test was then performed to identify the most significant features, and 6 of them with a *p* value less than 0.05 that best classified D^+^ and D^−^ were selected. The final drug-feature matrix, which includes 40 drugs and 6 significant features, is presented in Table S3 of the Supplementary materials.

#### Random forest classifier

**Figure 4:**
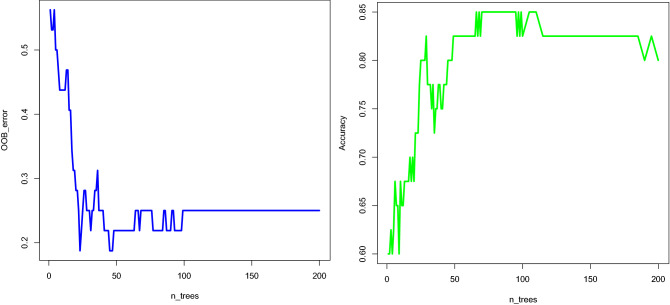
There is a trade-off Between OOB error and accuracy. One by one, trees were added to the algorithm, and OOB error and accuracy were calculated.

A random forest classifier based on the drug-feature matrix was used to construct a model for further analysis. The optimal number of trees was determined to be approximately 100 based on the tradeoff between OOB error and accuracy. Figure 4 depicts the findings.

Furthermore, fivefold cross validation (cv) was used to evaluate the constructed random forest classifier, and several performance measures were reported. During the evaluation 80% of the data was selected for the training dataset and 20% for the test dataset. As shown in Tables 6 and 7, the accuracy of each fold in a fivefold cv ranges between 0.75 and 0.875%, and the overall accuracy is 0.825. In addition, the precision and recall values for D^+^ and D^−^ range from 0.75 to 1. The F1 measures for negative and positive sets are 0.89 and 0.86, respectively. Results have been shown in Tables 6 and 7.

**Table 6:** Performance measures of the constructed random forest model with fivefold cv.

Fold 1 Acc	Fold 2 Acc	Fold 3 Acc	Fold 4 Acc	Fold 5 Acc	Overall Acc
0.875	0.75	0.875	0.875	0.75	0.825

*Accuracy: Acc.

**Table 7:** Confusion matrix.

	Precision	Recall	F1-score
Negative	0.8	1	0.89
Positive	1	0.75	0.86

#### Selecting relevant FDA approved drugs

**Figure 5:**
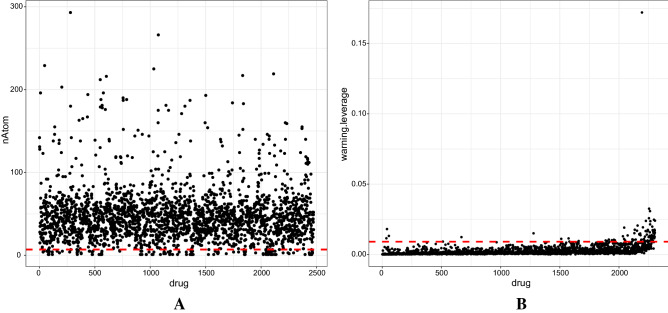
This figure depicts the D_FDA_'s outlier detection process. (**A**) Those with a small number of atoms (nATOM = 7) were initially removed from the dataset (drugs below the red dashed line). (**B**) The warning leverage was then calculated using the applicability of domain, and outlier drugs were excluded (drugs above the red dashed line).

The structural files of 2474 FDA-approved drugs (D_FDA_) were downloaded from DrugBank, and 1D-2D features were extracted from PaDEL. Drugs with a nATOM (number of atoms) less than 7 were eliminated (Fig. 5A). The 6 significant features obtained in the previous step were chosen for the remaining 2317 drugs. However, for 13 drugs, the value of some selected features was unavailable, so they were eliminated. The total number of drugs was thus reduced to 2304. To detect outliers, first, the applicability of the domain was determined, and then the warning leverage was computed as follows:

$${\text{Warning}}\;{\text{leverage}} = 3{\text{p}}/{\text{n}} = 3*\left( {6 + 1} \right)/2304 = 0.0091$$

Each drug with a warning leverage equal to or exceeding the aforementioned threshold was omitted (Fig. 5B). Consequently, 94 drugs were eliminated.

The remaining 2210 drugs were classified using a one-class classification SVM method, and 1221 drugs (D′_FDA_) were selected as the most relevant to at least one of the 40 drugs.

Then, D′_FDA_ was given as input into the constructed random forest model, and the probability of each drug belonging to the D^+^ or D^−^ was calculated. The drugs with a probability of 0.95 or higher were deemed the most relevant. Due to their positive effect on the regulation of lncRNAs associated with DM and AD, certain D^+^ drugs have been proposed as repositioning candidates. Those in D^−^ have been classified as adverse drugs as they have a negative effect on the regulation of corresponding lncRNAs. Table 8 lists all selected FDA-approved drugs discovered in this study.

**Table 8:** Final selected FDA approved drugs with positive and negative effects.

Positive drugs	Probability	Negative drugs	Probability
Dexamethasone	1	Panobinostat	1
Sorafenib	1	Diamorphine	1
Estradiol	1	Deferasirox	1
Fluorouracil	1	Nilotinib	1
Curcumin	1	Rosiglitazone	1
Quercetin	1	Trovafloxacin	1
Tamoxifen	1	Bisphenol A	1
Fulvestrant	1	Bexarotene	1
Diclofenac	1	Oxaliplatin	1
Chromium nicotinate	0.97	Vincristine	1
Tapentadol	0.97	Doxycycline	1
Niacin	0.96	Azathioprine	1
Chlorphenesin	0.95	Decitabine	1
Mephenesin	0.95	Etoposide	1
Homosalate	0.95	Verapamil	1
Sibutramine	0.95	Epirubicin	1
2-Mercaptobenzothiazole	0.95	Bortezomib	1
		Azacitidine	1
		Dihydro-alpha-ergocryptine	0.99
		Cefepime	0.99
		Epicriptine	0.984
		Alectinib	0.98
		Dihydroergocristine	0.974
		Deferoxamine	0.974
		Glipizide	0.974
		Ziprasidone	0.97
		Betrixaban	0.97
		Remimazolam	0.97
		Glimepiride	0.97
		Dihydroergotamine	0.97
		Tazemetostat	0.97
		Zaleplon	0.96
		Cocarboxylase	0.96
		Sulfasalazine	0.954

The final FDA-approved negative and positive drugs are listed in this table. Positive drugs are suggested for repurposing. Negative drugs, on the other hand, have been introduced as warning drugs that may disrupt the expression of common lncRNAs and significantly increase the negative effects of both diseases simultaneously.

The final FDA-approved negative and positive drugs are listed in this table. Positive drugs are suggested for repurposing. Negative drugs, on the other hand, have been introduced as warning drugs that may disrupt the expression of common lncRNAs and significantly increase the negative effects of both diseases simultaneously.

## Discussion

In this work, using network and machine learning approaches, candidate drugs for repurposing were introduced based on common mRNAs, miRNAs, and lncRNAs associated with the cross-talk between DM and AD. Drugs that may exacerbate the negative effects of both diseases were also discussed. The first 11 significant lncRNAs, as listed in Table 1, are taken into account. Only four of these lncRNAs have been associated with drug interactions in lncRNA-drug databases: NEAT1, XIST, H19, and MALAT1. The interactions with the remaining seven lncRNAs have not yet been identified. Therefore, this study focuses on the four lncRNAs listed above in order to identify suitable drugs for the repositioning objective.

Scientists believe that inhibiting NEAT1 and XIST reduces Aβ plaques in patients' brains by regulating the expression of interacted ncRNAs and proteins in specific pathways. Therefore, silencing of the aforementioned lncRNAs may represent a novel potential therapeutic target in the treatment of both diseases^44,45^. Furthermore, researchers show that inhibiting H19 prevents apoptosis in hippocampal neurons in both diseases^46^. Additionally, experiments indicate that overexpression of MALAT1 increases the lncRNA's neuroprotective function in the brains of patients and decreases neuronal injury in the hippocampus^47^.

The discovery of drugs that can silence NEAT1, XIST, or H19 while also inducing MALAT1 overexpression could be considered a new treatment for DM and AD cross-talk.

Conversely, drugs that have a negative effect on the targeted lncRNAs must be avoided. According to Table 8, there are two categories of FDA-approved drugs. Positive and negative drugs are listed, respectively, in the first and third columns. Positive drugs have been recommended for diabetic and Alzheimer's patients. Estradiol, for instance, is effective in preventing Aβ plaques and tau hyperphosphorylation in the brains of Alzheimer's patients, in addition to reducing the risk of diabetes^48,49^. It has been demonstrated that drugs in the chromium family have a curable effect on DM, AD, and IR, as they not only improve cognitive function in older adults but also regulate glucose excretion in patients with DM and IR^50^. Consequently, it may be one of the most significant candidate drugs in this cross-talk. Tapentadol, a drug used to treat pain in the elderly, is another positive drug. According to researchers^51^ it may improve the behavior of elderly dementia patients. Additionally, it has been proposed as a novel therapy for diabetic neuropathy^52^. Niacin is a B vitamin that facilitates the transformation of food into energy. According to research on the effect of niacin on AD, scientists believe that including niacin in one's diet can aid in disease prevention.^53^. Volunteers who received less niacin were up to 70 percent more likely to develop the disease than those who received more. However, it has the potential to increase glucose levels in diabetic patients, so dosage must be closely monitored^54^. Sibutramine is used for weight loss. Additionally, it reduces LDL cholesterol, thereby reducing the cardiovascular risk in diabetic patients^55^. Moreover, since high cholesterol levels may increase the risk of AD^56^, it may be a new drug capable of curing Alzheimer’s patients, particularly by controlling APOE.

Curcumin, a bright yellow chemical produced by curcuma, and quercetin, a plant pigment found in grapefruit, onion, and other fruits and vegetables, are two positive natural compounds. According to numerous studies, curcumin plays an important role in reducing Aβ plaques and inhibiting tau aggregation, as well as controlling blood sugar and managing diabetes complications. In addition, quercetin reduces oxidative stress by regulating signaling pathways, as well as lowering hyperglycemia and improving IR. In AD, it also has neuroprotective properties.

On the other hand, negative drugs have not been suggested for patients with DM and AD.

Diamorphine, for instance, increases tau hyperphosphorylation in AD patients by targeting the FYN protein^57^. The FDA has not approved zaleplon, a sleeping pill, for Alzheimer's patients because this class drugs have been linked to an increased risk of dementia in the elderly^58^. The anti-inflammatory drug, sulfasalazine, is used to treat Crohn’s disease and rheumatoid arthritis. Despite being an anti-inflammatory, chou et al. discovered that it may increase the risk of dementia in patients with rheumatoid arthritis. Hypoglycemia has been added to the list of side effects of sulfasalazine in T2DM patients^59,60^. Geodon is the brand name for the antipsychotic drug ziprasidone, which is used to treat schizophrenia and bipolar disorder. Experimental studies indicate that it increases the risk of death in Alzheimer's patients, so it is neither recommended nor approved for dementia patients^61^. Furthermore, it increases the risk of hyperglycemia and diabetes. Cefepime, an antibiotic used to treat bacterial infections, has been linked to disruption of brain function; therefore, Alzheimer's patients should only take it on a doctor's advice.

In DM or AD, drugs such as cocarboxylase, glipizide, glimepiride, and the ergotamine family are being investigated. They are, however, classified as negative drugs in our study because they have an adverse effect on the regulation of common lncRNAs, and further experimental research into their impact on DM and AD is recommended.

There is insufficient information regarding the associations between the remaining drugs and the intended diseases; therefore, more research is required.

In addition to the obtained drugs, we investigated the impact of well-known drugs on the expression of the significant mRNAs, miRNAs, and lncRNAs that are currently used for the treatment of DM and AD from DrugBank database. There are 52 and 11 DM and AD drugs, respectively. In our results, the anti-diabetic drug Rosiglitazone is categorized as a negative drug. Based on our findings, the use of this drug may increase the risk of AD by elevating NEAT1 expression (see Tables 4, 5). Therefore, this drug may be an important candidate for future experimental research on the role of anti-diabetic drugs in the progression of AD.

However, there is no other overlap between drugs that we introduced in this study and the remaining 62 DM or AD drugs in the DrugBank database.

In future work, we will increase the reliability of our study by evaluating the expression of introduced lncRNAs using human samples. Then, we intend to conduct a laboratory experiment at the Royan Institute for Stem Cells to test introduced candidate drugs on the obtained lncRNAs using mouse samples. On the other hand, we proposed that dysregulation of certain mRNAs and miRNAs causes cross-talk between DM and AD. Since the focus of this paper was on lncRNA-drug repurposing, one of the future studies could concentrate on drug repurposing based on introduced mRNAs and miRNAs.

This study can be expanded to include any other diseases with a correlation, such as DM and fatty liver or AD and Parkinson's disease. The pipeline remains unchanged; only the extracted data changes. Consequently, this work can serve as a guideline for similar work on two other diseases that interact with each other.

## Conclusion

Drug repurposing is one of the most important methods for developing new drugs from existing ones for the treatment of diseases. This paper proposed a network and machine learning-based method that not only suggests drugs to inhibit DM and AD cross-talk, but also introduces drugs that exacerbate the negative effects of the aforementioned diseases.

For instance, the drugs chromium nicotinate and tapentadol, which are used to treat IR and pain, respectively, may regulate the expression of NEAT1, XIST, MALAT1, and H19 in the brains of diabetic and Alzheimer's patients. Consequently, these two drugs can be considered a novel treatment for this cross-talk. In addition, drugs such as ziprasidone and sulfasalazine, which are used to treat crohn's disease and schizophrenia, may increase the risk of both DM and AD. The list of repurposing candidate drugs and adverse drugs for which we wish to conduct experimental research to confirm our findings is presented in Table 8. Two of the drugs in this table, curcumin and quercetin, are natural compounds that can regulate the expression of H19, XIST, and MALAT1. Therefore, long-term use of these natural products may reduce the risk of DM and AD.

## Supplementary Information

Supplementary Information 1.

Supplementary Information 2.

Supplementary Information 3.

## Data Availability

All data generated or analysed during this study are included in this published article [and its supplementary information files].

## Abbreviations

IR: Insulin resistance
DM: Diabetes mellitus
AD: Alzheimer’s disease
T3D: Type 3 diabetes
APOE: Apolipoprotein E
IDE: Insulin degradation enzyme
GLUT4: Glucose transporter type 4
AChE: Acetylcholinesterase
T2DM: Type 2 diabetes mellitus
NcRNA: Non-coding RNA
MiRNA: MicroRNA
LncRNA: Long non-coding RNAs
CADD: Computer aided drug design
FDA: Food and Drug Administration
OOB: Out of bagging error
SVM: Support vector machine
CV: Cross validation
Aβ: Amyloid beta
